# Microindoline 581, an Indole Derivative from *Microbacterium* Sp. RP581 as A Novel Selective Antineoplastic Agent to Combat Hepatic Cancer Cells: Production, Optimization and Structural Elucidation 

**DOI:** 10.22037/ijpr.2020.111982.13469

**Published:** 2020

**Authors:** Roya Pournejati, Ronald Gust, Brigitte Kircher, Hamid Reza Karbalaei-Heidari

**Affiliations:** a *Molecular Biotechnology Laboratory, Department of Biology, Faculty of Science, Shiraz University, Iran. *; b *Department of Pharmaceutical Chemistry, Institute of Pharmacy, Center for Molecular Biosciences Innsbruck, University of Innsbruck, CCB–Centrum for Chemistry and Biomedicine, Innrain 80-82, 6020 Innsbruck, Austria. *; c *Immunobiology and Stem Cell Laboratory, Department of Internal Medicine V (Hematology and Oncology), Innsbruck Medical University, Anichstrasse 35, 6020 Innsbruck, Austria. *; d *Tyrolean Cancer Research Institute, Innrain 66, 6020 Innsbruck, Austria.*

**Keywords:** DNA damage, Microbacterium sp., Microindoline, Natural products, Hepatocellular carcinoma

## Abstract

Screening of bioactive compounds with potential binding affinity to DNA as one of the target molecules in fighting against cancer cells has gained the attention of many scientists. Finding such compounds in the cellular content of microorganisms, especially marine bacteria as valuable and rich natural resources, is of great importance. *Microbacterium* sp. RP581, as a member of *Actinobacteria* phylum, was isolated from the Persian Gulf coastal area and the production of the target compound was optimized using statistical methods in cheap culture ingredients. The purification of the target compound was performed by flash chromatography and preparative HPLC. Both molecular and structural analyses indicated that the compound was an indole derivate which was tentatively named as Microindoline 581. Interaction of Microindoline 581 with genomic and circular DNA revealed that this compound can cause double- strand breaks through binding to the DNA. The analysis of cellular growth and proliferation of various cancer cell lines suggested proper and specific effect of the Microindoline 581 towards HepG2 cells with an IC_50_ of 172.2 ± 1.7 µM. Additional studies on cell migration inhibition and cell-death induction indicated a concentration-dependent inhibitory effect on proliferation and induction of death of HepG2 cells. The selective action of Microindoline 581 which was isolated from the *Microbacterium* sp. RP581 in killing HepG2 cells might be due to its specific metabolism in those cells as a precursor.

## Introduction

Natural products have historically been considered as a rich source of therapeutic agents and continue to play an extremely important role in the development of therapeutics. Structures of almost 200,000 natural products have been introduced and each year about 4,000-5,000 novel compounds are characterized and added to the list (1, 2). Reports indicate that approximately 60% of successful drugs are from natural sources (3), which demonstrate the importance of this area of research. Among the diverse sources of natural producers, marine bacteria constitute an infinite pool of novel chemistry, making up a valuable source for discovery and identification of bioactive compounds. In particular, strains belonging to the order of *Actinobacteria* are Gram-positive filamentous bacteria that account for more than 45% of all bioactive metabolites discovered in nature (4). Over the past century, the therapeutic use of antibacterial natural products, such as Actinomycin D, Daunorubicin, Mitomycin, Tetracycline and Vancomycin has had an intense impact on human health (5). The discovery of promising anticancer candidates including Salinosporamide A and Bryostatin only intimates the remarkable wealth of marine bacteria as potential drug producers (6, 7).

Apart from discovering novel bioactive metabolites, optimizing the production conditions and enhancing bioavailability coupled with lowering the costs are highly desirable from biomedical applications point of view. To do so, selection of low cost carbon, nitrogen sources as well as optimizing the culture conditions through fractional factorial design and response surface methodology (RSM) are common practices and researchers have focused on statistical approaches for the production of bioactive metabolites (8, 9). 

On the other hand, nowadays cancer is one of the leading causes of death worldwide which is expected to increase rapidly as populations grow. Moreover, age, and lifestyle behaviors increase cancer risk (10). The latest estimates on the global burden of cancer have risen to 18.1 million new cancer cases (17.0 million except for non-melanoma skin cancer) and 9.6 million cancer deaths (9.5 million except for non-melanoma skin cancer). In both sexes, lung cancer is the most commonly diagnosed cancer (11.6% of the total cases) and the leading cause of cancer death (18.4% of the total cancer deaths), closely followed by female breast cancer (11.6%), prostate cancer (7.1%), and colorectal cancer (6.1%) for incidence, and colorectal cancer (9.2%), stomach cancer (8.2%), and liver cancer (8.2%) for mortality (11-13). Hepatocellular carcinoma (HCC) is the third cause of cancer-related death in both male and female populations. Multiple treatment alternatives exist for patients with HCC including curative resection, liver transplantation, radiofrequency ablation and systemic chemotherapy. The therapeutic use of anticancer drugs is limited due to severe side effects caused by their poor selectivity toward cancerous cells relative to non-neoplastic ones. Moreover, HCC has acquired resistance to many drugs (14, 15). So, searching for new selective anticancer agents with low systemic toxicity is an urgent need.

In the present work, we succeed to isolate *Microbacterium* sp. RP581, which belongs to *Actinobacteria *phylum, from Persian Gulf short line in Khorshahab village port, Bushehr province, Iran. There are few reports on producing a cytotoxic metabolite from *Microbacterium *including the production of Microbacterins A and B by *Microbacterium sediminis *sp. nov. YLB-01 (T) (16), levans by *Microbacterium levaniformans* (17), glycoglycerolipids by sponge associated *Microbacterium* sp. (18) and the organic extract of *Microbacterium mangrovi* MUSC 115^T^ isolated from Mangrove forest soil, Malaysia (19). In this report, we describe a bioactive compound identified from a newly isolated *Microbacterium* sp. RP581, which is particularly toxic on hepatocellular carcinoma cells, probably via DNA binding activity. Culture medium components were selected from low-cost sources. The optimization of different variables, which affected the bioactive compound production, was performed using statistical approaches. The bioactive compound was partially purified, and its anticancer properties were evaluated using MTT, crystal violet, colony forming, and scratched assay as well as flow cytometry test.

## Experimental

*Materials*

Chemicals were provided either from Merck or Sigma-Aldrich. All the solvents were purchased from Samchun Company Ltd., Republic of Korea with the appropriate grade. The human red blood cells were gifted from the Blood transfusion organization of Fars providence, Shiraz, Iran. SV-80, U-2-OS and HT29 cell lines were purchased from cell line service (CLS) GMbH, Eppelheim, Germany. U86, A549, MCF-7 ad HepG2 cells were obtained from Department of Cell Bank, Pasture Institute of Iran.

*Bacterial isolation and genus identification*

Sediment samples were collected from 50-cm depth in Khorshahab village coastal area of the Persian Gulf (Figure S1). The samples were processed immediately after collection using the selective method of dilution and heat-shock treatment, followed by inoculating onto the isolation medium, M1. The dilution and heat-shock method were carried out as described by Mincer *et al*. (20). Briefly, 1 mL of each wet sediment was added to 4 mL of sterile seawater and heated for 40 min at 55 °C while vigorously being shaken. They were further diluted (1: 4) in sterile seawater and then 150 μL of each diluted sample was inoculated through spreading onto agar-based isolation medium using a sterile glass rod.

The isolation medium (M1) consisted of the following ingredients: 10 g of starch, 4 g of yeast extract, 2 g of peptone, 18 g of agar, reached up to 1 L by natural seawater followed by autoclaving. Afterward, it was amended with filtered cycloheximide (100 μg/mL) and Kanamycin (20 μg/mL) in order to avoid growth of fungi and Gram-negative bacteria, respectively. For the isolate genus identification, 16S rDNA gene of the isolate was amplified by PCR (21). Amplified fragments were purified from 1% agarose gel and sequenced. The 16S rDNA sequence data were analyzed by MEGA7.0.26 software, and the phylogenetic tree was constructed using the neighbor-joining algorithm (22).

*Optimization procedures and experimental design*

*selection of carbon and nitrogen sources*

Six different media supplying 10 g/L of three low cost carbon sources (wheat flour, baking starch and molasses) along with two inorganic nitrogen sources (5 g/L (NH_4_) _2_SO_4_ and 10 g/L KNO_3_) were designed and the media were supplemented with 1 g/L yeast extract as vitamin source. Inoculation was done through transferring 300 µL of a 3 d seed culture (in Tryptic soy broth (TSB) medium) into a 100 mL Erlenmeyer flask containing 30 mL of the above mentioned media. Cultivations were conducted at 30 °C and 180 RPM for 3 d. All experiments were performed in triplicate.

*Influence of environmental factors on secondary metabolite production using fractional factorial experiments*

For selection of the most significant variables affecting the bioactive compound production, five variables (X1/wheat flour, X2/(NH_4_)_2_SO_4_, X3/seawater, and X4/pH, X5/inoculation size) were tested and analyzed by the fractional factorial (2^5-1^) design experiment. The principal effects of each variable on the value of bioactive compound production were represented at the high and the low levels. The experimental design with the variables, symbol codes, and experimental levels of the variables are shown in Supporting data, (Tables S1) and (Table 1), respectively. The bioactive compound production value (response) was calculated by the following equation:

Response (%) = Cell toxicity (%) × Relative organic extract production

Where “Relative organic extract production” is the amount of the organic extract (mg/mL) in a culture condition/highest amount of organic extract (mg/mL) in the experiment.

*RSM design*

Response surface methodology (RSM) was applied to identify optimum levels of more effective variables including wheat flour (X_1_), (NH_4_)_2_SO_4 _(X_2_) and seawater (X_3_) to reveal maximum response percentage. The coded independent variables used in the RSM design are listed in (Table S3) (Supporting data). The experiments were designed according to the central composite design (CCD) using a 23 factorial and star design with six central points, as shown in (Table 2). Data were analyzed by Design Expert software 7.0.0. 

*Purification procedure*

The isolate RP581 was cultured in the optimized medium at 30 °C and 180 RPM for 3 d and biomass was collected by centrifugation at 9000 g, for 10 min. The biomass was then treated and shaken with an equal volume of acidic methanol (1: 24) for 45 min, three times. Methanol was evaporated using rotary evaporator. For active compound extraction, the initial crude methanolic extract was washed by chloroform and its cytotoxicity was tested by above-mentioned normalization as response percentage. The chloroformic fraction (3.0 g) was subjected to flash chromatography (silica gel 60, Merck) with ethyl acetate/ethanol gradient (100:0 to 90:10) to yield five fractions. The fraction No # 5 (60 mg) was purified via semi-preparative HPLC (C18, TSK gel 7.8 × 300 mm) through running a linear gradient of H_2_O/MeOH (30:70 to 0:100) for 20 min, followed by running for 25 min with 100% MeOH at 1 mL/min, and detection wavelength of 254 nm. Afterwards, the structural properties of the active compound were elucidated by FTIR, ^1^H NMR and HR-MS. Infrared (IR) spectra were recorded using an Alpha FT-IR Spectrometer (Bruker, Billerica, USA).^ 1^H-Nuclear magnetic resonance spectra (^1^H-NMR spectra) were recorded using Bruker Avance II 600 spectrometer (Bruker Biospin, Rheinstetten, Germany) in CD_3_OD. Chemical shifts are given in parts per million (ppm) and the coupling constants are given in Hertz (Hz). High resolution mass spectrometry (HR-MS) was performed using an Orbitrap Elite (Thermo Fisher Scientific, Waltham, USA) in negative mode.

*Microindoline 581–DNA interaction study*

*Fluorescence spectroscopy*

Fish DNA stock solution was prepared in PBS buffer and concentrations of the DNA solutions were determined by using the average extinction coefficient value of 6600 M^-1^ cm^-1^ at 260 nm. The ratio of UV absorbances at 260 and 280 nm (A_260_/A_280_) greater than 1.7 indicates that the DNA was sufficiently free from protein. The emission changes of Microindoline 581 (90 µM) at 450 nm (excited at 254 nm) were monitored by addition of increasing amount of DNA (0 µM to 172 µM) at room temperature. The Stern-Volmer plot was drawn and the binding constant was calculated by the following equation (23):

I_0_/ I = 1 + (ε_Q_[Q]/ε_B_[B]) × (1+ K_SV_[Q])

Where F_0_ and F are the fluorescence emissions (λ = 447) in the absence and presence of the quencher, respectively. Q and B are the concentration of quencher (DNA) and binder (microindoline 581), respectively. The K_sv_ stands for the Stern Volmer constant, and the molar extinction coefficient of the quencher and the binder are denoted by ε_Q_ and ε_B_, respectively. 

*Gel electrophoresis*

The DNA structural changes induced by Microindoline 581 was analyzed using gel electrophoresis technique. Briefly, circular pBluescript KS (+) plasmid DNA was incubated with different concentrations of Microindoline 581 in PBS buffer (pH ~ 7.4) at 37 °C for 1 h under both physiological and oxidative (50 µM H_2_O_2_) conditions. Mechanistic studies were done using different additives (NaN_3_ 100 mM; DMSO 100 μM; KI 100 μM and EDTA 100 μM) prior to addition of the bioactive compound (24). The samples were then analyzed by 1% agarose gel electrophoresis applying tris-acetic acid-EDTA (TAE) buffer (pH ~ 8.2) at 50 V for 50 min. The gel was stained with 0.5 μg/mL ethidium bromide and visualized by UV light and photographed for analysis. The cleavage efficiency was measured by determining the ability of the Microindoline 581 to convert the supercoiled (SC) DNA (Form I) to the nicked circular (NC) form (Form II) and/or the linear circular (LC) form (Form III). 

*Inhibitory effect on DNA replication*

Polymerase chain reaction (PCR) was performed to study the inhibitory effect of Microindoline 581 on DNA replication using *Thermoanerobacter thermohydrosulfuricus* lipase (TtL) gene as template. Template DNA was incubated with different concentrations of the compound at 37 °C for 1 h, then amplification reaction of the 700 bp fragment was carried out in 50 µL reaction solution using appropriate forward and reverse primers (25).

*Hemolytic activity*

Fresh human red blood cells (RBC) were rinsed and suspended in PBS to obtain an A600nm (OD600) of 24 and added to the tubes followed by further incubation with Microindoline 581 in a final concentration of 500 µg/mL, 1% methanol 1% and 1% Triton-x100 as negative and positive controls respectively at 37 °C for one hour. Then, the mixture was centrifuged at 2000 g for 10 min. The supernatant absorbance at 450 nm was measured using SPECTROstar® Nano (BMG, Germany). Experiments were performed in triplicates. Hemolysis percentage was calculated by using the following equation:

Hemolysis percentage = (A_450_ sample/A_450_ control) × 100

*Cell culture*

Cancerous cells (HepG2 (hepatocellular carcinoma), MCF-7 (breast cancer), A549 (lung adenocarcinoma), U86 (glioblastoma), HL60 (acute promyelocytic leukemia), HT29 (colorectal adenocarcinoma), U2-OS (osteosarcoma), and SV80 (immortalized fibroblast cell line) were maintained in a humidified atmosphere containing 5% CO_2_ at 37 °C in Dulbecco’s modified eagles medium (DMEM) supplemented with 2 mM L-glutamine, 100 units mL^−1^ penicillin, 100 μg mL^−1^ Streptomycin and 10% fetal bovine serum (GIBCO, USA). The cells were serially passaged twice a week.

*MTT assay*

A cell viability assay was performed using the MTT method. Briefly, exponentially growing cells were seeded into 96-well flat bottom plates and incubated for 24 h at 37 ℃ in the presence of 5% CO_2_. Then, a defined number of cells were exposed to the active compound, Microindoline 581, in different concentrations from 15 to 600 μM for a period of 48 h. Afterwards, the MTT (3-(4,5-dimethylthiazol-2-yl)-2,5-diphenyltetrazolium bromide) solution was added to achieve a final concentration of 0.45 mg mL^-1^ which was further incubated for 4 h at 37 °C. Finally, equal volume of solubilization solution (40% (V/V) dimethyl formamide (DMF) in 2% (V/V) glacial acetic acid and 16% sodium dodecyl sulfate (SDS), pH ~ 4.7) was added to each well to dissolve formazan crystals. The absorbance was recorded at 570 and 630 nm (turbidity assessment) using the SPECTROstar^®^^Nano^ microplate reader (BMG LABTECH, Germany). Cell viability percentage was calculated by the following equation:

Cell viability % = [A_T__(sample)_/A_T (control)_] × 100

A_T_ = A_570 _- A_630_

*Crystal violet assay*

HepG2 cells were treated with various concentrations of Microindoline 581 for 48 h. After aspirating the media, the cells were washed with pre-warmed PBS and fixed with 0.25% glutaraldehyde solution for 30 min at room temperature. The cells were washed with PBS and stained with 0.02% crystal violet solution for 30 min at room temperature followed by washing with tap water for 5 times to remove the extra crystal violet. Finally, 180 µL of ethanol (70%) was added to each well to dissolve the crystal violet. The absorbance was recorded at 570 nm using the SPECTROstar^®^^Nano^ microplate reader. Average OD570 of the control (non-stimulated) cells was set to 100% and the percentage of the treated cells that are viable (attached) was determined by using the following equation:

Cell viability (%) = (the average OD570 values of the samples /the OD570 values of the control cells) × 100

*Colony forming assay*

HepG2 cells were treated with different concentrations of Microindoline 581 for 48 h. Then, the cells were trypsinized and seeded (200 cells/well) in a 6 well plate and left for 14 days in the incubator. The cells were washed twice with PBS and fixed with methanol for 20 min at room temperature. They were washed twice again with deionized water and stained with 1% crystal violet for 10 min. Finally, they were further washed with deionized water for 5 times, and then air dried. The colonies were counted under an optical microscope.

*Scratch wound healing assay*

HepG2 cells were plated and grown to confluence in 24 well plates to form a monolayer culture. Then, the cell layer in each well was scratched using a 10 µL pipet tip and once the scratch was made, medium was removed and replaced with fresh medium supplemented with 90 µM of Microindoline 581 and incubated for 48 h. Images were taken after the scratch was induced and also 24 and 48 h after the Microindoline 581 administration. The migration distance was quantified with Image J software and the migration rate calculated by equation:

Cell migration rat (%) = (scratched distance (initial time) – scratched distance (after 48h)/scratched distance (initial time) × 100

*Cell-death analysis*

Apoptosis/necrosis induction on HepG2 cells by Microindoline 581was determined with flow cytometry. Briefly, the cells were seeded in a 6-well plate with a density of 2 × 10^5 ^cells/well and incubated for 24 h. Then, the culture medium was replaced by fresh medium, which contained different concentrations of Microindoline 581 (90, 147, and 172 µM), and the cells were further incubated for 24 h. Culture media and the cells were collected and the experiment was proceeded according to the manufacturer’s instruction of FITC Annexin V apoptosis detection kit. The analysis was performed within an hour by flow cytometry (FACS Canto II, Becton Dickinson). The cells were discriminated into necrotic, early apoptosis, late apoptosis, and living cells using FLOWJO 10.5.0 software.

*Detection of morphological changes*

6 x 10^6^ cells were adjusted to 1 x 10^6^ cells/mL and placed in a 12-well plate followed by incubation at 37 °C in a humidified 5% CO_2_/95% air atmosphere for 24 h to let the cells adhere. Thereafter, 160 µM Microindoline 581 was added and the cells were cultivated for another 72 h. A picture was taken by JuLITM Live cell imaging system (NanoEnTek, Seoul, Korea) every 1 h.

## Results

*Bacterial isolation and identification*

Several marine bacteria were isolated from Persian Gulf short line coasts and evaluated for production of cytotoxic compounds toward hepatocellular carcinoma cells (HepG2) (26). Amongst them, a *Microbacterium* was selected as the potent candidate to produce active metabolites to kill hepatocellular carcinoma cells and was chosen for further study. *Microbacterium* sp. RP581 (GenBank ID: KU217326), belonging to the *Actinobacteria* superfamily, is a coccus, Gram-positive, spore forming, catalase-positive, coagulase-negative, and oxidase-negative bacterium with ability to ferment glucose, fructose, sucrose, dextrose, and mannitol (Figures S1 and S2). 

*Microindoline 581 production optimization using statistical approach*

**Figure 1:**
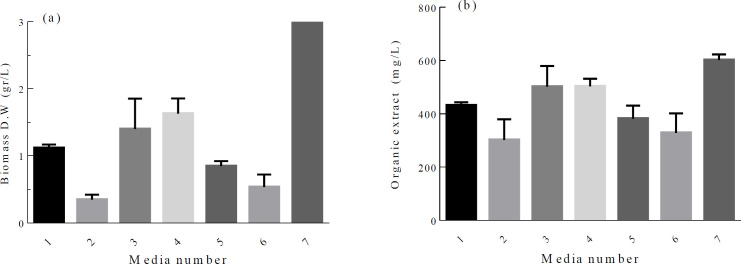
Carbon and nitrogen source selection experiment.* Microbacterium* sp. RP 581 biomass and the organic extract production with respect to media number

Secondary metabolism usually occurs at the late growth phase of microorganisms. The ability to produce secondary metabolite has certainly genetic background, but gene expression and target compound production can be affected significantly by environmental manipulations. Among the nutrients, the effect of carbon and nitrogen sources on bioactive compound production has been studied by many researchers (27, 28). In our work, firstly, a matrix of different carbon and nitrogen sources was designed. Cell growth and crude organic extract content vary in different culture media. Although maximum cell growth was recorded when baking starch and ammonium sulfate were used, the highest ratio of crude organic extract*/*biomass and the lowest IC_50_ value of 4.35 ± 0.27 mg/mL were obtained when wheat flour and ammonium sulfate were used as carbon and nitrogen sources, respectively (Figure 1). Based on this data, a set of 2^5-1 ^fractional factorial experiments were conducted to study the effect of 5 independent variables (Table S1). Experimental results for 2 level fractional factorial design showed significant variation in the calculated response with a range of 0.56 to 88.9% (Table 1). The best (run 16) and the lowest (run 1) responses were documented when all the variables adjusted at the + 1 level, and at the-1 level, respectively, indicating that all the variables have positive effect on the response.

Analyzing the data by Design Expert software 7.0.0 revealed that, while the wheat flour, ammonium sulfate and sea water concentrations were the most significant parameters, the inoculation size and pH had little effect on the response value (Table S2). For further optimization of bioactive compound production, the three significant factors were selected and the spherical central composite design (CCD) was conducted at five different levels (Table 2 and Table S3). A 14 factorial design and six axial points (α = 1.73) with six replicates at the central point were designed to yield a total of 20 experiments. On analyzing the data using regression, a second-order polynomial equation was obtained to express the responses as a function of the independent variables as follows:

Response = 78.37 + 6.82 X_1_ + 8.88 X_2_ – 13.94 X_3_ - 4.49 X_1_X_2_ – 9.25 X_1_X_3_ – 10.27 X_2_X_3_ - 14.70 X_1_^2^ - 15.36 X_2_^2^ - 14.82 X_3_^2^

**Figure 2:**
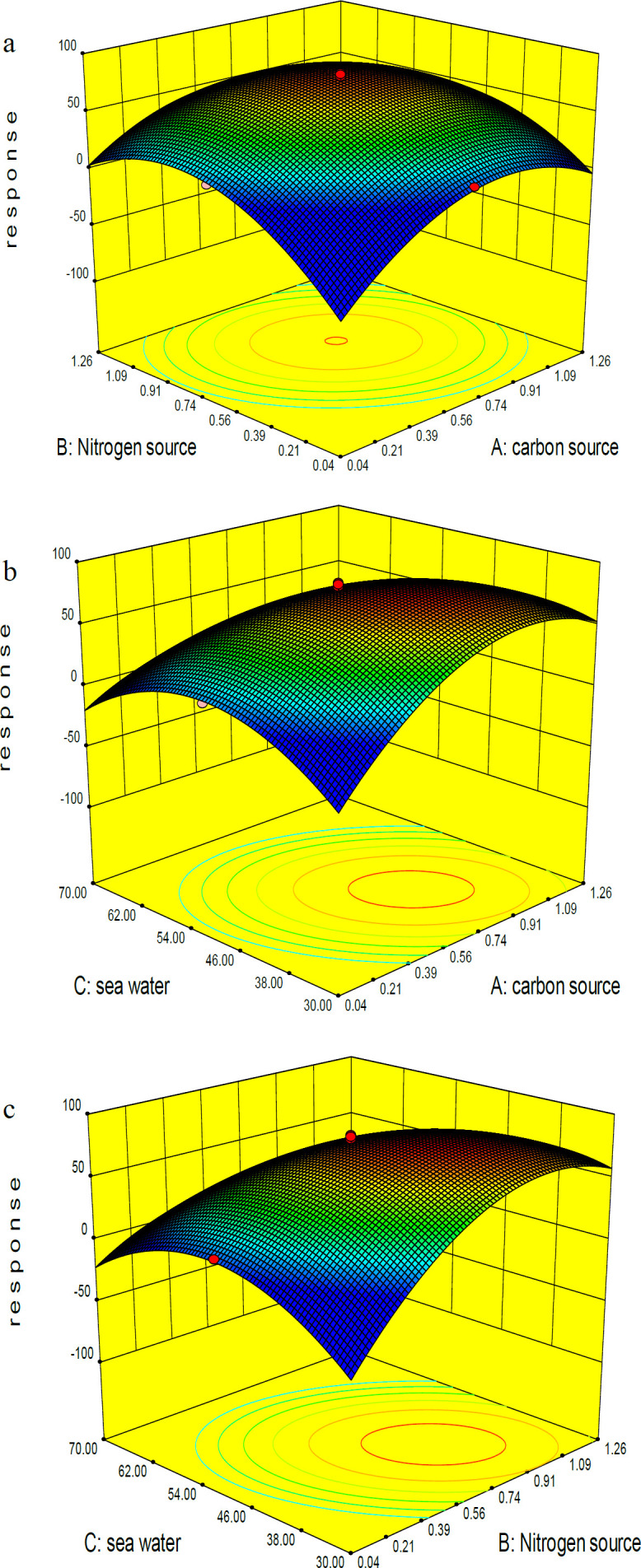
3D graph of three independent variables and their interaction in RSM experiments

In terms of actual factors, the obove equation can be written as:

Response = -218.41 + 265.40 A +285.65 B + 4.82 C – 36.68 AB -1.32 AC – 1.46 BC -120.00 A^2^ -125 .42 B^2^ – 0.04 C^2^

where A, B, and C represent wheat flour, ammonium sulfate and sea water concentrations, resectively. The model term is strongly significant at 99% confidence level (*P*-value ˂ 0.05) with a R^2^ value of 0.977 indicating flat curves with the best condition in maximal points of the selected levels for all significant variables. The F value of 47.41 implies that there is only a 0.01% chance that this model could occurs due to noise. X_1_, X_2_, X_3, _and the interaction between them are significant in this study (Table S4). Comparative analysis of three significant parameters revealed that positive effect of wheat flour and ammonium sulfate concentrations on the bioactive compound production was stronger than the negative effect of sea water concentration run 1, 4, 5 in (Table 2). Increasing or decreasing the wheat flour and ammonium sulfate concentrations to 1.26% and 0.04% resulted in reducing the response. Comparison of the response recorded for runs 9, 10, 11 and 12 also confirmed the above conclusion about the positive effects of wheat flour and ammonium sulfate concentrations: 19.38%, 44.89%, 17.65%, and 40.64%, respectively. The dissident trend was observed in the case of sea water (runs 13 and 14: 49.93% and 11.61%). Regarding the predicted model, a composition of 0.65% wheat flour, 0.65% ammonium sulfate, and 50% sea water can lead to the best response runs 15-20, (Table 2). (Figure 2) shows the 3D graphs regarding the interactions between the three independent variables and confirms the optimization of all three components in the central point of coded values.

*Microindoline 581 purification and characterization*

**Figure 3:**
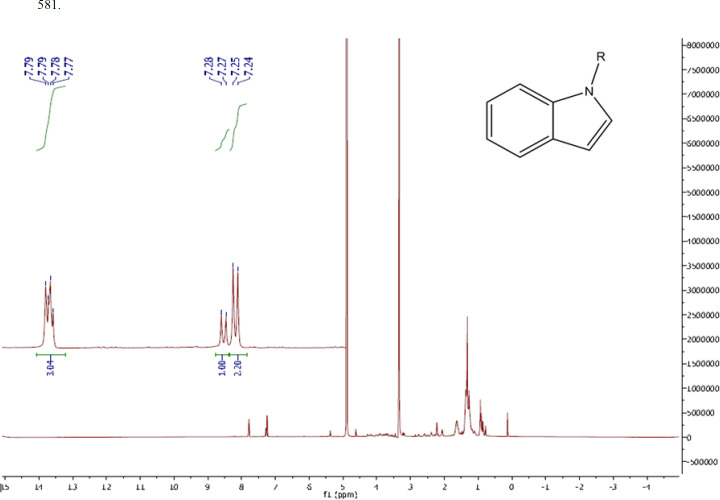
^1^H NMR spectra and proposed structure of Microindoline 581

As mentioned in the methods section, the active compound (Microindoline 581) was purified from chloroformic fraction of the crude extract of *Microbacterium* sp. RP581 by combination of flash column chromatography and preparative HPLC approaches. The purification procedure was started with 3.0 g of the crude extract loaded on a flash chromatography column. The target compound was found in fraction E (EtAc 9.5: EtOH 0.5) and was further purified by reversed-phase HPLC to yield 1.3 mg of the active compound. The negative mode ESI-MS data indicated a molecular mass of 340 Da (Figure S3a). The FTIR spectra (Figure S3b) revealed the presence of aromatic and aliphatic C-H bonds (841.32 and 2854.08 cm^-1^, respectively), C = C bonds (1449.57 cm^-1^), benzene ring (2926 and 1617 cm^-1^), C-N bonds (1113.95 cm^-1^) as well as N-H, C-H, and O-H bonds (3415.44 cm^-1^). The ^1^H-NMR signals (600MHz; CD_3_OD) at δ (ppm) = 7.24(d, 2H, 2CH, J = 7.8Hz), 7.73-7.8 (d, 2H, 2CH, J = 8.4 Hz), (d, 1H, CH, J = 7.8 Hz) indicated the presence of an indole core (Figure 3). Two peaks at δ (ppm) = 3.0 and 5.0 are related to the solvent. Therefore, based on structural data analysis, the compound was tentatively named Microindoline 

*DNA interaction and cell toxicity study*

**Figure 4:**
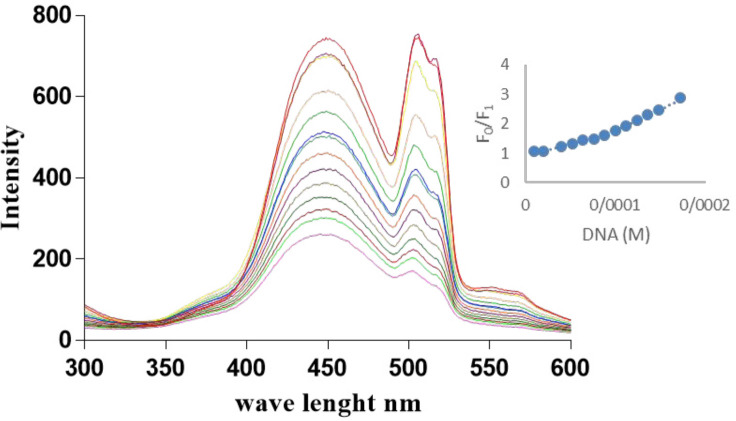
Fluorescence spectra of Microindoline 581 and F-DNA interaction. The Microindoline 581 (90 µM) was incubated with a range of F-DNA concentration (0-172 µM). The Stern –Volmer plot of binding was drawn

The DNA binding kinetics and properties were evaluated by titration of Microindoline 581 with fish DNA using fluorescence spectroscopy. As shown in (Figure 4), by increasing the DNA concentration, a decrease in the fluorescence emission was observed, indicating that the compound was intercalated in DNA structure. A non-linear upward Stern–Volmer plot (Figure 4) is a result of competition in light absorption mechanism (23). In this mechanism, the quencher also absorbs the excitation light (λ = 260) and consequently, an upward curvature is observed in S-V plot. 

**Figure 5:**
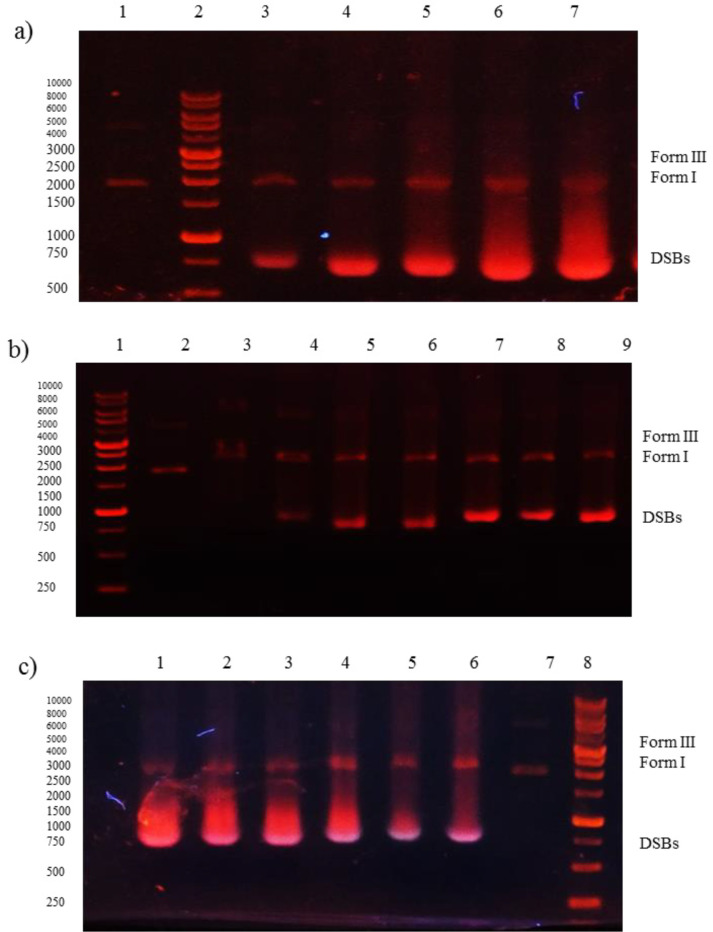
Gel electrophoresis (1% agarose) of plasmid DNA treated with Microindoline 581; a) lane 1, DNA + H_2_O_2_ (50 µM) (negative control), lanes 3-7, DNA + H_2_O_2_ (50 µM) treated with 1.5 mM, 2 mM, 2.5 mM, 3 mM and 6 mM of Microindoline 581, respectively, at 37 °C for 1 h; b) lane 2, DNA + H_2_O_2_ (50 µM) (negative control), lanes 3-9, DNA + H_2_O_2_ (50 µM) treated with 300 µM of Microindoline 581, 1 h, 3 h, 6 h, 9 h, 16 h, 20 h and 24 h, 37 °C respectively; c) lane 1, DNA + H_2_O_2_ (50 µM) + 1.5 mM of Microindoline 581, lane 2, DNA + H_2_O_2_ (50 µM) + 1.5 mM of Microindoline 581 + 100 µM of DMSO, lane 3, DNA + H_2_O_2_ (50 µM) + 1.5 mM of Microindoline 581+ 100 µM of KI, lane 4, DNA + H_2_O_2_ (50 µM) + 1.5 mM of Microindoline 581 + 100 µM of EDTA, lane 5, DNA + H_2_O_2_ (50 µM) + 1.5 mM of Microindoline 581 + 100 µM of NaN_3_, lane 6 DNA + 1.5 mM of Microindoline 581 and lane 7, DNA + H_2_O_2_ (50 µM) (negative control)

The K_sv_ was estimated as 3.25 × 10^3^ M^-1^ with R^2 ^= 0.98, which indicates the strong interaction with dsDNA. Furthermore, the ability of Microindoline 581 to mediate DNA damage was assessed using the pBlueScript II KS (+) plasmid and applying 1% agarose gel electrophoresis (Figure 5). As (Figures 5a and 5b) indicate, the Microindoline 581 can induce DNA break in a concentration- and time-dependent manner. As shown in (Figure 5a), the DNA double strand breaks (DSBs) was induced under oxidative conditions (50 µM H_2_O_2_, similar to physiological concentration in cancerous cells) after 1 h of treatment with at least 1.5 mM Microindoline 581 (29). When the concentration of Microindoline 581 reduced to 300 µM, the circular DNA cleavage was observed after 3 h of incubation. This experiment was extended to 24 h. By increasing the time of incubation, the severity of double-strand breaks in DNA molecule increased significantly (Figure 5b). Moreover, the role of oxidative stress induced by H_2_O_2 _and ROS species, involved in DNA cleavage was investigated by addition of ROS chelators as described in the experimental section (Figure 5c). Reduction of DSBs occurrence in the presence of NaN_3 _demonstrated the role of singlet oxygen (^1^O_2_) in the cleavage induced by Microindoline 581. To investigate the inhibitory effect of Microindoline 581 on the DNA replication, polymerase chain reaction (PCR) was conducted. The data revealed the inhibitory effect of Microindoline 581 on amplification of target DNA in a concentration-dependent manner (Figure S4). 

**Figure 6:**
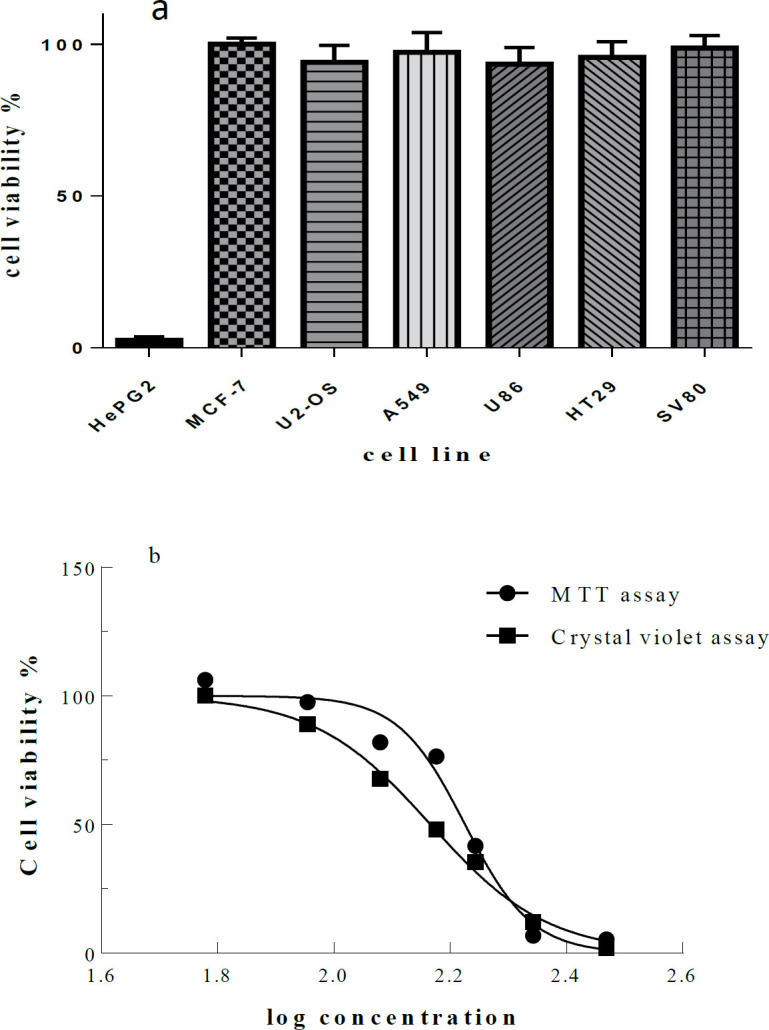
**.** )a) Cell viability determined by MTT assay. Various cancer cell lines were treated with Microindoline 581 (600 µM) for 48 h. The mean viability ± STD of three experiments is shown. Cellular viability in the absence of the compound was set at 100% and) b) Cellular viability of HepG2 cells. Cell viability percentage was determined by MTT and crystal violet assay after treatment with various concentrations of Microindoline 581 for 48 h

The Microindoline 581 was tested in proliferation and cytotoxicity assays against the cancerous cell lines and fibroblast cell line SV80 as normal control. Interestingly, Microindoline 581 was selectively active against HepG2 cells. Even at a concentration of 600 µM Microindoline 581, no toxicity induced in the other cell lines (Figure 6a). Almost identical results were obtained by MTT and crystal violet assay indicating an IC50 value of 172.2 ± 1.7 µM and 146.2 ± 2.5 µM to decrease viability of HepG2 cells when incubated for 48 h, respectively (Figure 6b).

**Figure 7:**
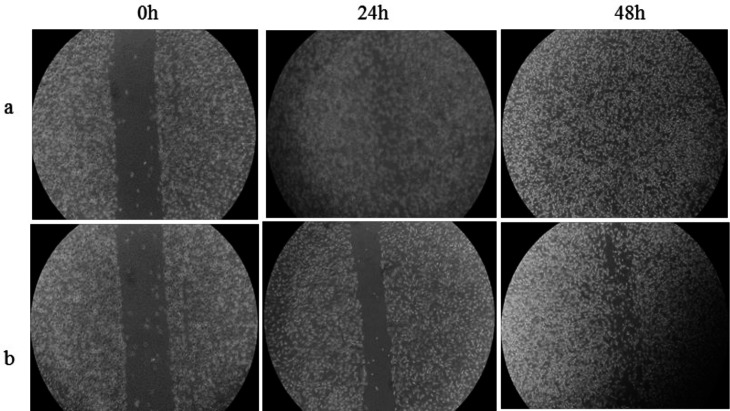
HepG2 cells migration analysis using scratch assay )a) Control group, )b) HepG2 cells treated with 90 µM of Microindoline 581

The ability of the DNA repair system to remedy DNA damage in the treated cells and chronic cytotoxicity was measured using colony forming assay (Figure S5, and Table S5). The IC_50 _value after 48 h treatment of the cells by Microindoline 581 was calculated to be 83.5 ± 1.6 µM. Moreover, as shown in )Figure 7), a cell migration inhibition assay using the scratch method revealed reduction of cellular proliferation and proper migration when treated by the Microindoline 581 (90 µM) compared to the control group (without the compound). The cell migration rates were calculated as 42.75 and 71.52% after 24 and 48 h treatment, respectively. 

Finally, the ability of Microindoline 581 to induce cell death in HepG2 cells was investigated using Annexin V and PI staining and analysis by flow cytometry. After 24 h of treatment, the percentage of necrotic cells and apoptotic cells were increased in a concentration-dependent manner, whereby a strong reduction of viable cells was observed with higher concentrations (147 and 172 µM, respectively) (Table 3). Additionally, the morphological changes observed after addition of Microindoline 581 (90 µM) underlined its cytotoxic behavior against HepG2 cells. As shown in (Figure S6), HepG2 cells showed epithelial-like morphology (polygonal in shape with more regular dimensions) at the beginning of the treatment but after 72 h, they appeared rounded which indicates cell death occurred. Finally the hemolytic activity of Microindoline 581 was assessed on human RBCs. Only 8.2 ± 2.4% hemolysis was observed when RBCs treated with Microindoline 581 in 200 µM final concentration.

## Discussion

In the field of screening new anticancer agents, marine bacteria have been recognized as rich and promising resources, continuously producing novel therapeutic compounds, which contribute significantly toward chemotherapy (30). We conducted a marine bacterial library isolated from Persian Gulf, Iran. Among various isolates, *Microbacterium* sp. RP 581 was selected for further investigation. The Microindoline 581 was purified from *Microbacterium* sp. RP 581. It shared the same basic indole structure with the other compounds, reported from *Microbacterium* sp. MCCC1A11207 including novel Microindolinone A which showed no cytotoxic effect when tested on RBL-2H3 cells (31). The indole moiety is one of the promising ring systems for drug development and has been termed as “privileged structure” (32). The capacity of >NH group for hydrogen binding, the high π-electron, and the highest occupied molecular orbital (HOMO) energy of the planar indole ring are the reasons why the indole interact with nucleobases through groove binding or π-π stacking (33). The DNA binding properties of indole derivatives are reported in literature (34, 35). Indole derivatives show different bioactivities including antibacterial, cytotoxic, and antineoplastic potential and have significant values in pharmaceutical development (36). Hepatocellular carcinogenesis is the result of genetic alteration affecting several signaling cascades, mainly, overexpression in multiple key proteins including vascular endothelial growth factor (VEGF), epidermal growth factor, Ras mitogen activating protein kinase (MAPK), insulin-like growth factor receptor, hepatocyte growth factor/c-MET, I3K/PTEN/Akt/mammalian target of rapamycin (mTOR), and Wnt/ß-catenin pathways which are various ways to guide cell toward uncontrolled proliferation (37-39). Consequently, chemotherapeutic agents need to either block one or more steps in a targeted pathway to achieve suitable response. Moreover, the acquired constant resistance to standard first-line systemic drug for advanced HCC results in limited benefits (40). The clinical significance of DNA-binding compounds can hardly be ignored, as many anticancer regimens include a compound that binds to and/or modifies DNA such as Doxorubicin; but the poor selectivity toward tumor cells relative to non-neoplastic ones limited its application. So, chemo agents that target unique characterizations of cancerous cells in their mechanism of action are highly desirable. Accordingly, the compounds which interact with or affect DNA via mechanisms involved H_2_O_2_ can act as specific drugs to treat cancer with (41, 42). As shown in (Figures 4) and 5, Microindoline 581 interacts and intercalates into DNA and consequently induces dsDNA breaks in a concentration- and time-dependent manner. Moreover, our data demonstrated that the DSBs increased in the presence of H_2_O_2_ which indicated the specificity of Microindoline 581 toward cancerous cells. Interestingly, Microindoline 581 only affected the viability of HepG2 cells (Figure 7). Albeit, DNA interaction is a general mechanism of action; DSBs induction was observed at much higher concentrations compared to cellular toxicity assays. It seems that a special metabolization was occurred on the Microindoline 581 in HepG2 cells which resulted in production of a more active compound on DNA structure and showed a selective toxicity on this type of cancer cells in lower dosages.

**Table 1:** Experimental results for 2 level fractional factorial design

**Response**	**Cell Death (%)**	**X**_5_	**X**_4_	**X**_3_	**X**_2_	**X**_1_	**Run**
0.65	0.89 ± 1.47	+1	-1	-1	-1	-1	1
5.17	4.99 ± 2.12	-1	-1	-1	-1	+1	2
24.74	38.3 ± 16.9	-1	-1	-1	+1	-1	3
82.02	91.07 ± 0.68	+1	-1	-1	+1	+1	4
23.2	48.51 ± 10.35	-1	-1	+1	-1	-1	5
71.49	81.62 ± 15.34	+1	-1	+1	-1	+1	6
31.29	95.04 ± 0.43	+1	-1	+1	+1	-1	7
50.06	61.06 ± 15.66	-1	-1	+1	+1	+1	8
21.75	28.48 ± 5.42	-1	+1	-1	-1	-1	9
1.2	2.13 ± 1.4	+1	+1	-1	-1	+1	10
8.53	13.33 ± 3.33	+1	+1	-1	+1	-1	11
53.45	87.81 ± 5.86	-1	+1	-1	+1	+1	12
42.46	91.16 ± 3.66	+1	+1	+1	-1	-1	13
69.91	91.51 ± 5.07	-1	+1	+1	-1	+1	14
8.45	11.94 ± 2.17	-1	+1	+1	+1	-1	15
88.89	88.9 ± 6.012	+1	+1	+1	+1	+1	16

**Table 2:** The spherical central composite design (CCD) including percentages of “Cell death”, “Response” and “Predicted response”.

**Predicted response (%)**	**Response (%)**	**Cell death (%)**	**X**_3_	**X**_2_	**X**_1_	**Run**
7.72	9.22	15.64 ± 3.55	-1	-1	-1	1
48.84	52.96	98.53 ± 1.73	-1	-1	+1	2
55.54	62.45	97.34 ± 2.51	-1	+1	-1	3
78.16	83.08	98.45 ± 1.63	-1	+1	+1	4
18.88	18.64	40.32 ± 4.56	+1	-1	-1	5
21.04	20.24	23.51 ± 4.85	+1	-1	+1	6
25.08	25.63	50.98 ± 3.54	+1	+1	-1	7
11.24	14.4	32.8 ± 1.95	+1	+1	+1	8
22.62	19.38	27.05 ± 2.01	0	0	-1.73	9
46.22	44.89	64.52 ± 1.1	0	0	+1.73	10
21.83	17.65	37.24 ± 4.87	0	-1.73	0	11
43.05	40.64	70.32 ± 0.2	0	+1.73	0	12
58.18	49.93	49.93 ± 14.24	-1.73	0	0	13
9.94	11.61	16.21 ± 7.32	+1.73	0	0	14
78.37	73.42	99.24 ± 0.1	0	0	0	15
78.37	75	95.41 ± 4.27	0	0	0	16
78.37	80.43	97.31 ± 2.67	0	0	0	17
78.37	83.36	97.45 ± 3.39	0	0	0	18
78.37	75.74	97.07 ± 2.03	0	0	0	19
78.37	82.24	97.45 ± 3.02	0	0	0	20

**Table 3:** Cell death induction in HepG2 cells treated with Microindoline 581 for 24 h

**Concentration (µM)**	**necrotic cells**	**late apoptotic cells**	**early apoptotic cells**	**alive cells**
0	2.95 ± 1.05	10.05 ± 0.05	2.55 ± 0.25	84.35 ± 1.15
90	7.95^***^ ± 1.05	10.80 ± 1.70	2.20 ± 0.30	79.05^*^ ± 0.35
147	4.9^**^ ± 1.7	15.6^**^ ± 0.80	13.15^**^ ± 4.25	66.35^**^ ± 1.75
172	22.55^***^ ± 4.65	8.45 ± 1.55	1.75 ± 0.05	67.25^**^ ± 3.05

*p-*value* < 0.05, ** *p*-*value* < 0.005, *** *p*-*value* < 0.0005.

## Conclusion

In the present study, we identified an indole derivative, named Microindoline 581, isolated from *Microbacterium* sp. RP581 which induces double strand breaks in DNA in the presence of H_2_O_2_ (50 µM) *in-vitro*. Moreover, Microindoline 581 is selectively toxic against HepG2 cells, as shown by MTT and crystal violet assays. The wound-healing effect of the compound in the cell migration inhibition assay also confirmed its potent effect as drug. According to its safety behavior in blood circulation, as seen in the hemolysis activity test, it can be introduced as a good candidate for further investigation for HCC therapy.
